# Comparing international dementia research priorities—Systematic review

**DOI:** 10.1002/gps.5836

**Published:** 2022-11-03

**Authors:** Monica Logan, Stephanie Leitch, Zainab Bosakh, Lucy Beishon, Terence J. Quinn

**Affiliations:** ^1^ School of Medicine University of Glasgow Glasgow UK; ^2^ Department of Cardiovascular Sciences University of Leicester Leicester UK; ^3^ Institute of Cardiovascular and Medical Sciences University of Glasgow Glasgow UK

**Keywords:** dementia, global healthcare, priority setting, research

## Abstract

**Objectives:**

Research priority setting aims to collate stakeholder opinion to determine the most pressing research questions. Priority setting exercises influence decisions around research funding, development and policy. We compared published dementia research priority setting exercises from international healthcare systems.

**Methods:**

Four multidisciplinary, international, electronic databases were searched for relevant studies (2010 until 2021). Priorities were extracted, coded and assigned to categories using thematic analysis. The Nine Common Themes of Good Practice (9CTGP) and the Reporting guideline for priority setting of health research (REPRISE) checklists were used to assess methodological and reporting quality respectively.

**Results:**

From 265 titles, 10 priority setting exercises (1179 participants, 147 priorities) were included. Studies spanned four continents and the majority included people living with dementia and their care‐givers in the priority setting process (68%). Only one paper met all the best practice indicators. Issues around inclusiveness, implementation and evaluation of the priorities were apparent in nine papers. We categorised priorities under eight themes: caregivers (25%, *n* = 37), support (24%, *n* = 35), awareness and education (16%, *n* = 24), drugs and interventions (14%, *n* = 21), diagnosis (8%, *n* = 12), pathology (6%, *n* = 9), research design (5%, *n* = 7), and prevention (1%, *n* = 2). Priorities varied by geographical region, with awareness and education of higher priority in low‐middle income countries, compared to caregivers and support in high income countries.

**Conclusions:**

Key priorities were identified with some commonality around themes considered of greatest importance. There is scope to improve the process and reporting of priority setting. Priorities differed according to contextual factors and so, priorities specific to one healthcare setting may not be applicable to others.

## Key points

- Priority setting exercises identify the most pressing research questions and are used to shape the research and funding agenda.
- Caregivers, support, and awareness and education were the top three dementia research priorities.
- Priorities varied by healthcare setting with awareness and education highest ranked amongst low‐middle income countries compared to caregivers and support amongst high income countries.
- Future attempts at prioritisation need to pay particular attention to how the priorities will be implemented and evaluated.

## 1 INTRODUCTION

Dementia is a global burgeoning public health crisis.^1^ Research may provide useful answers to the many problems associated with the growing prevalence of dementia. Internationally, dementia research is gaining visibility and creating impact, as highlighted by the G8 Dementia Summit and 2012 World Health Organisation report on dementia.^2^, ^3^ However, to capitalise on the potential power of dementia research we need to ask the right questions.

The evidence base for dementia research remains weak compared to certain other chronic conditions,^4^, ^5^ perhaps, in part, because dementia research remains relatively underfunded, receiving eight times less than cancer research.^6^, ^7^ With a limited resource, and a seemingly infinite number of research questions, there is need to find equitable methods to support research activity. The dementia research space is characterised by strongly held and sometimes apparently conflicting views from dementia experts and professionals. It could be argued that we are more likely to determine the research questions that are most meaningful to people living with dementia if we involve them in the research process. Creating a consensus on the most important topics for future research would help progress the research agenda.

Formal research prioritisation exercises offer a potential approach to achieving this consensus. The prioritisation process seeks to draw on the opinions of all stakeholders, including professionals, caregivers, advocates and people with lived experience.^8^ Decisions are made around which are the most pressing research questions and subsequent lists of priority research topics can be used to target resource accordingly.^8^, ^9^ Research prioritisation is increasingly used by funding bodies and charities to direct calls for research or inform the assessment of applications for research funding.

**FIGURE 1:**
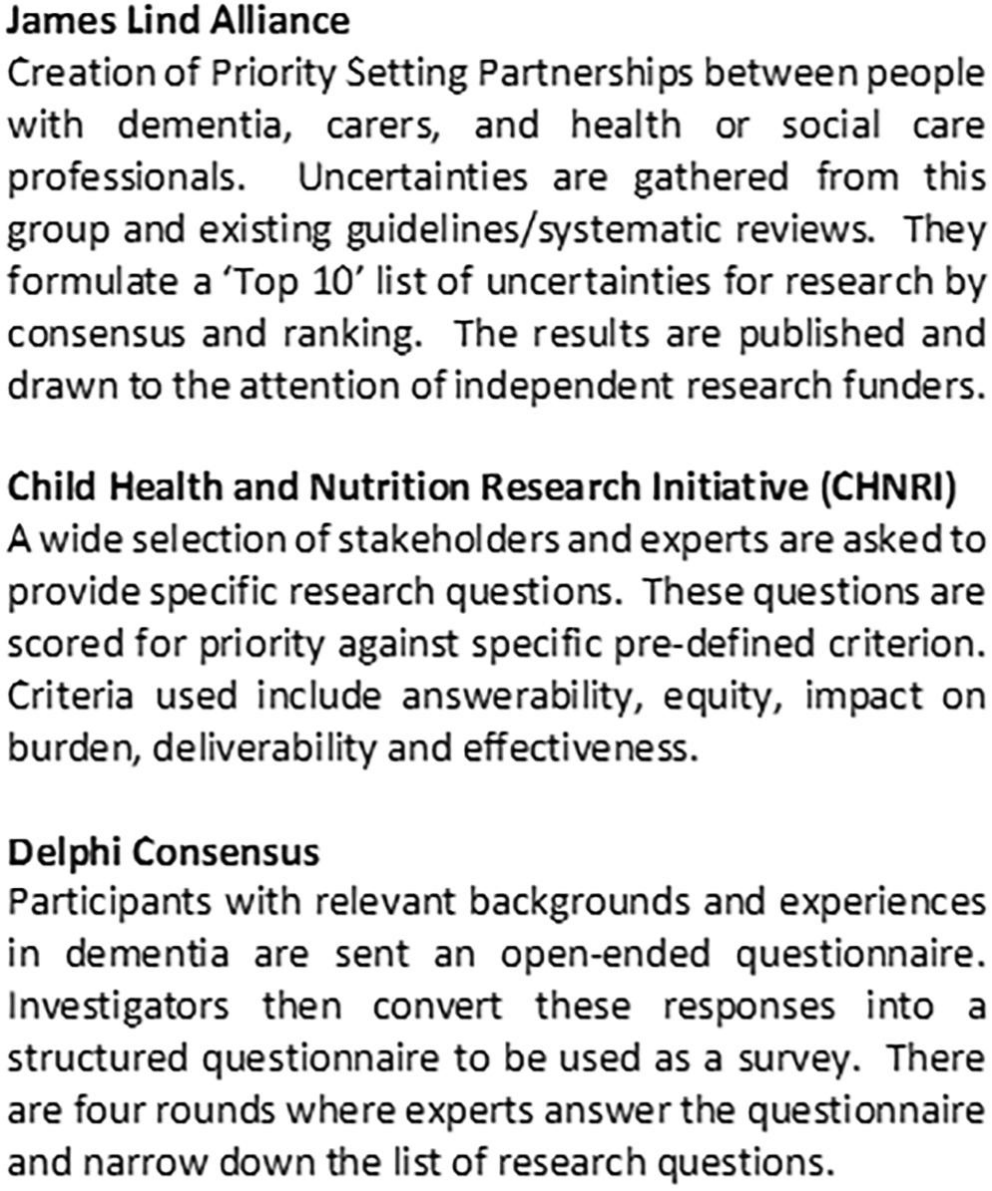
Description of the three major priority setting methods used in research prioritisation.

There are three common methods for conducting research prioritisation exercises: the Child Health and Nutrition Research Initiative (CHNRI) method, the Delphi method and the James Lind Alliance method.^10^, ^11^, ^12^, ^13^ A full description of each of these methods is provided (Figure 1), but all share common features of stakeholder involvement with ranking and consensus agreement on the top‐rated priorities. Best practice in the method and reporting of research prioritisation has been described and tools for assessing conduct and reporting are available.

Dementia research priorities may vary by context, for example, low‐middle income countries with fewer resources, and younger populations, are facing different dementia related challenges to higher income areas.^14^ Priorities may also vary according to the mix of stakeholders who participated in the process, or the processes that were followed.

Therefore, the aim of this review was to collate, and compare published dementia research prioritisation exercises and determine if there was agreement on common research priorities. Secondly, we aimed to examine how research priorities were moderated by geography, stakeholder demographics and conduct of the priority setting exercise.

## 2 METHODS

### 2.1 Search strategy and inclusion criteria

This review was reported according to the Preferred Reporting Items for Systematic Reviews and Meta‐Analyses (PRISMA) guidelines.^15^ We did not pre‐register a protocol for this review. MEDLINE (OVID), EMBASE (OVID), CINAHL (EBSCO) and PsycINFO (EBSCO) databases were searched systematically from 2010 until 2021. This time frame was chosen to reflect contemporary priorities, as we recognised that dementia research, health and social have changed substantially since the early 2000s. We created search terms based on key concepts of ‘dementia’ and ‘research prioritisation’ using validated research syntax for both. The full search strategy can be seen in Supporting Information S1. In addition to database searching we searched websites of key organisations involved in prioritisation or dementia research: James Lind Alliance, Alzheimer's Association, Alzheimer's Research UK, Alzheimer's Society and World Health Organisation.

All aspects of screening, title selection, data extraction, quality assessment and synthesis were performed independently by two trained reviewers (ML, SL) who compared results. Discrepancies were resolved by discussion with recourse to a third party (TQ) as needed. Inclusion criteria were: (1) published paper with a process that was described as prioritisation (2) focus on dementia (any subtype) (3) presents a list of priorities, (4) published in English or German. Figure S1 summarises the PRISMA flow diagram for studies included in this review. Data extraction sheets were developed and piloted on two exemplar papers by a single researcher (ZB).

### 2.2 Quality assessment

Included studies were assessed for methodological and reporting quality using two validated tools: The Nine Common Themes of Good Practice (9CTGP) provides a nine‐item checklist to assess methodological quality, while the Reporting guideline for PRIority Setting of health research (REPRISE) assesses quality of reporting against 32 items, organised under 10 domains.^16^

We graded studies using a traffic light system. The results of the quality assessments were not used to exclude any of the priority setting exercises. Instead, they were used to identify common areas of limitation or excellence across the published reports, and to determine the association between study quality and individual priorities. We used Cohen's kappa statistic calculated to measure inter‐rater reliability in the quality assessment scores assigned by the paired reviewers. One paper written in the German language was assessed for quality and reporting by a single native German speaker (SK) to minimise translation errors.

### 2.3 Data extraction and analysis

Each study reported a final list of top priorities, varying in number of priorities and detail. We extracted this final definitive list from each paper. We used the original text from each paper to collate a ‘long list’ of verbatim priorities and then categorise these using a thematic analysis approach. We created a preliminary set of categories based on initial recognition of common themes. Categories were re‐assessed and refined, adding or combining categories until all priorities were allocated, giving a final list of eight dementia research priority categories (Table 1). The number and percentage of identified research priorities are presented by category across the included studies. Initial agreement between the two researchers was assessed as percentage agreement and final allocation was based on a consensus. Figure S2 lists examples of priorities coded under each major category.

**TABLE 1:** Themed categories for priorities from evidence synthesis with their corresponding definition

Categories	Definition
Prevention	Prevention of dementia and understanding risk factors
Pathology	Understanding disease mechanisms, causes or stages of disease
Diagnosis	Role of identification of the disease and diagnostic tools
Drugs and interventions	Using drugs and other interventions to manage dementia (symptomatic treatments or disease modification)
Support	Supporting people with dementia in their daily life
Caregivers	Addressing the needs of caregivers, and how to support them
Awareness and education	Educating and raising awareness of dementia and dementia‐related issues for people living with dementia, care‐givers, lay public and professionals
Research methods	To improve the design, conduct, reporting and implementation of primary dementia research

## 3 RESULTS

**FIGURE 2:**
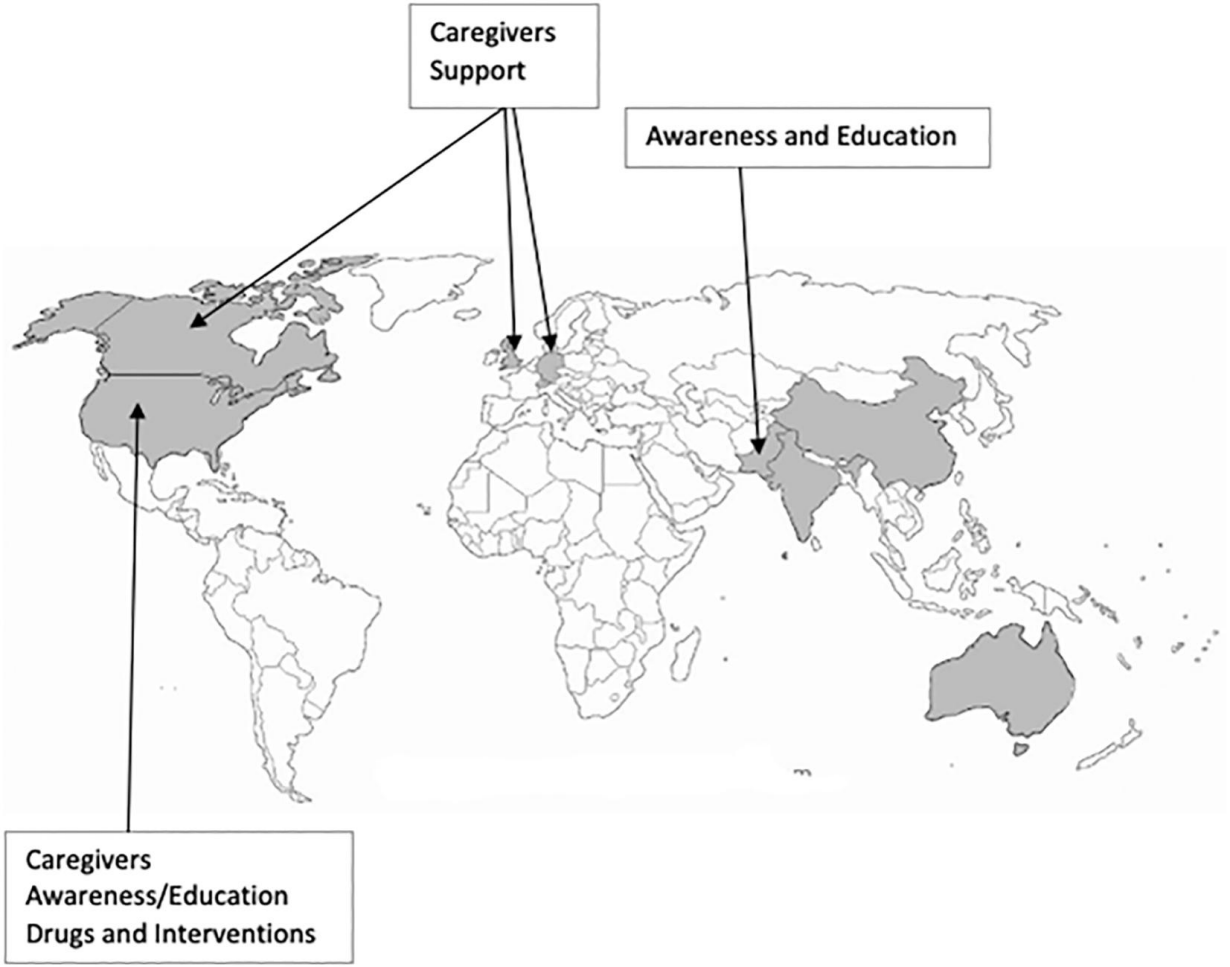
Global distribution of studies included in this review, and the top priorities highlighted by region.

From 265 titles, 11 full texts were reviewed, and 10 studies (1179 participants) were included in the final synthesis. Included studies came from a variety of countries (Figure 2), although the majority (*n* = 9) were in high,^17^, ^18^, ^19^, ^20^, ^21^, ^22^, ^23^, ^24^, ^25^ rather than low‐middle income (*n* = 2)^17^, ^26^ countries. Only one study was international in scope, including 39 countries (Europe, southeast Asia, Africa, the west Pacific, and the Americas).^17^

All of the three main prioritisation methods were employed: James Lind Alliance^19^, ^20^, ^22^ (*n* = 3, 30%), CHNRI^17^, ^26^ (*n* = 2, 20%) and the Delphi consensus method^23^, ^24^ (*n* = 2, 20%). People with dementia were included in seven studies,^17^, ^18^, ^19^, ^20^, ^21^, ^22^, ^23^ carers in eight studies,^17^, ^18^, ^19^, ^20^, ^21^, ^22^, ^23^, ^26^ and researchers/clinicians in six studies.^17^, ^20^, ^22^, ^24^, ^25^, ^26^ The majority of participants were patients and carers (801 participants, 68%), followed by clinicians and researchers (230 participants, 20%), representatives of the voluntary and third sector (39 participants, 3%), and other stakeholders (109 participants, 9%). Policy makers were only consulted in two studies.^17^, ^25^ Most studies were published within the last 4 years (*n* = 6). A description of the key characteristics and stakeholder groups within each paper are presented in Table 2.

**TABLE 2:** Data are number (percentage)

Name of paper	Country	Stakeholder groups (involved in final priority setting)						Methods	No. priorities identified
		People living with dementia	Care‐givers	Researchers	Clinicians	Third sector	Other		
Shah et al. 2016^17^	39 countries (Worldwide)	1	18	142		15	23	CHNRI	21
Leroi et al. 2019^26^	Pakistan	‐	6	15	11	4	4	Adapted CHNRI method	10
Armstrong et al. 2020^18^	USA	20	25	‐	‐	‐	‐	A semi‐structured questionnaire	9
Bethell et al. 2018^19^	Canada	7	5	‐	‐	5	11	JLA PSP	10
Kelly et al. 2015^20^	UK	2	5	‐	6	‐	5	JLA PSP	10
Law et al. 2013^21^	Scotland	404					65	Questionnaire	4
Enrich mills et al. 2019^22^	England	3	4	1	2	1	‐	Adapted JLA method	10
Hirt et al. 2020^24^	Germany, Switzerland, Austria	‐	‐	10	3	1	1	Delphi method	49
Porock et al. 2015^23^	USA	301		‐	‐	‐	‐	Delphi study‐modified snowball sample	6
Stolee et al. 2011^25^	Canada	‐	‐	17	23	13	‐	Consensus style focus group	18
Total (*n*, %)	1179	801 (68)		230 (20)		39 (3)	109 (9)		147

*Note*: A description of papers in the evidence synthesis and participants used in their priority setting exercises.

Abbreviations: CHNRI, Child Health and Nutrition Research Initiative; JLA, James Lind Alliance; PSP, priority setting partnership.

### 3.1 Priorities

**FIGURE 3:**
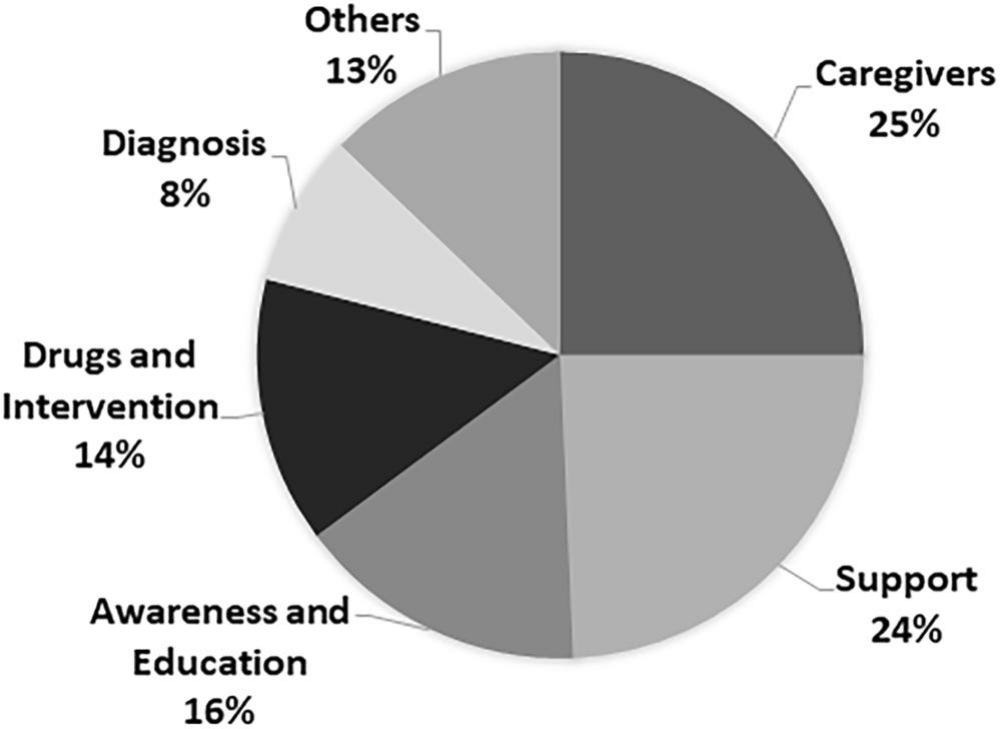
Percentage of priorities identified within each category. ‘Others’ comprises of: prevention, research design, and pathology.

A total of 147 priorities were identified from the 10 included studies. Range of priorities reported was 6–49. All priority items could be categorised under eight common themes. Agreement between the two researchers on priority categorisation was 82.7%. The majority of priorities focussed on the theme of ‘caregivers’ (*n* = 37, 25%), followed by ‘support’ (*n* = 35, 24%), ‘awareness and education’ (*n* = 24, 16%) and ‘drugs and interventions’ (*n* = 21, 14%). The categories containing the least number of priorities were: ‘diagnosis’ (*n* = 12, 8%), ‘research design’ (*n* = 7, 5%), ‘pathology’ (*n* = 9, 6%) and ‘prevention’ (*n* = 2, 1%). The latter three were collated into a section labelled as ‘others’. Priorities varied by geographical location, with high‐income countries identifying ‘caregivers’ and ‘support’ as their top two priorities, whereas low‐income countries identified ‘awareness and education’ as the top priority. Figure 3 summarises the proportion of priorities in each category.

### 3.2 Quality assessment

The completed 9CTGP and REPRISE checklists are summarised at study level in Table 3 and Table S1, respectively. In terms of methodological quality, context, relevant criteria, and transparency were well conducted across all studies. Where‐as, inclusiveness, implementation, and evaluation were poorly conducted across nine studies. Only one study fulfilled all areas of the checklist representing best practice.^17^ Agreement between the two assessors was 86.7% with a Cohen's kappa statistic of 0.73 (*p* < 0.005), indicating substantial agreement.

**TABLE 3:** Nine common themes of good practice checklist for each study included in the review^27^

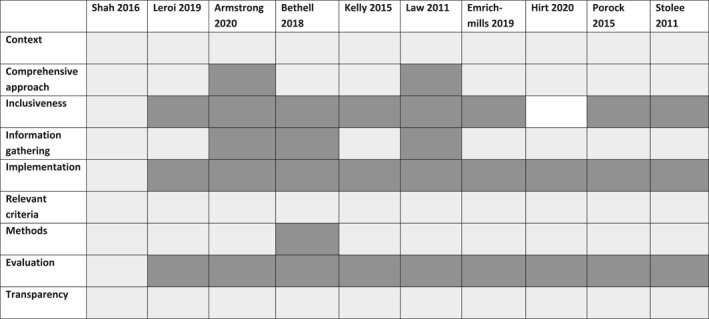

*Note*: Components marked light grey represent high quality practice whilst components marked in dark grey represent low quality practice. White indicates moderate quality.

In terms of reporting quality, no study fulfilled all the criteria in the REPRISE checklist. Only two studies achieved most areas (72%–75%) set out in the checklist.^17^, ^26^ Context and scope were generally well reported by studies (87%), where‐as evaluation and feedback (5%), implementation (30%), and funding/conflicts of interest (37%) were less well reported. None of the included studies reported the budget or described the approach to feedback priorities to stakeholders. The majority of studies used a framework for reporting priority settings (*n* = 7, 70%) with all studies stating how they engaged stakeholders and the number involved. The researchers agreed on 86.6% of the REPRISE checklist with a Cohen's kappa statistic of 0.734 (*p* < 0.005) indicating substantial agreement.

The top priority from the only study^17^ which met all the 9CTGP checklist was the delivery of care and services for people living with dementia and their carers. In comparison, the three studies^18^, ^19^, ^21^ with the lowest quality on the 9CTGP checklist found improving awareness,^17^ the impact of stigma,^19^ and early detection were the highest rated priorities.^21^

## 4 DISCUSSION

This review identified key priorities for the future dementia research agenda. The majority of published prioritisation exercises identified caregivers and support as the top priorities for dementia research, with lesser interest in drugs and treatment. These priorities seem to conflict with the traditional dementia research agenda, which has been focussed around identifying novel targets and disease modifying therapies.^28^, ^29^ This highlights the value of including the voices of people with lived experience of dementia in the prioritisation process. The published dementia research priority exercises included in this review are relatively recent, and it may be that they have not yet had time to have traction in the dementia research community. We hope that future dementia research activity will be more mindful of stakeholder priorities.

The high rating given to caregivers and support in this review is likely to be a reflection of the large representation of people living with dementia and their carers (68% of participants) in the stakeholder groups consulted. Involvement of patients and public in the planning of dementia research is a relatively recent phenomena.^30^, ^31^ Priority setting exercises have attempted to redress the perceived historical imbalance by weighting representation towards patients and carers. However, this risks under‐representation of other key stakeholder groups such as policy makers, third sector organisations and clinicians. Balanced representation across all potential stakeholders is the ideal. Interestingly, two studies included in this review,^24^, ^25^ with larger representation from researchers and clinicians, still identified caregivers as their top priority.

No study in our review included ‘at risk’ individuals for the development of dementia (e.g., healthy older adults, family history, genetic risk) as stakeholders. This likely explains the apparent low prioritisation for prevention based research. Studies included in this review only enrolled people living with milder forms of dementia. Involving people with moderate to severe dementia can be challenging and priority methods are often not conducive to their involvement (e.g., Delphi studies, focus groups).^32^ It seems plausible that priorities around more advanced dementia would shift towards issues of care‐giver strain, symptom management, and palliative care.^33^

In a recent scoping review of studies which analysed dementia care, key priorities for service improvement were person‐centred dementia care, integrated care pathways, and healthcare workforce development.^5^ The majority of these sources included views from European health and social care staff and commissioners. It would seem that dementia care priorities differ from the priorities seen in dementia research, but shared theme is the importance of considering the biopsychosocial aspects of dementia, rather than a purely biomedical view.

Compared to cancer, dementia attracts less research funding and has fewer successful therapies,^2^ which may result in differing research priorities. However, in a study examining the priorities of people living with and beyond cancer, diagnosis, support, and needs of caregivers were again highlighted as top priorities, consistent with the findings in this review.^34^ Notably, awareness and education was not a priority for people living with cancer, although this was identified as the third highest priority for dementia. This may suggest that public health campaigns around dementia need to be prioritised in the same manner that was used to raise public awareness of cancer and cancer risk factors.

Despite the anticipated growth in dementia amongst low‐middle income countries, only two studies^17^, ^26^ in this review examined research priority setting in these geographical areas. Research priorities differed by location, particularly between low‐middle and high income countries. Given the rapidly changing demographics in these countries, research priorities that are both current and relevant to low‐middle income countries are urgently needed. Our results suggest that we should not assume that a priority setting exercise conducted in one setting will be applicable or relevant to another setting.

We can use this review to inform future priority setting exercises. Future priority setting should consider, balanced representation across all key stakeholder groups, including at risk individuals, and people living with differing stages of dementia. We found substantial heterogeneity in the methods used and the reporting of results. This lack of standardisation makes comparisons between published research priorities problematic. Mapping to a standard template may negate this issue.^35^ Based on the results of our quality assessment, future studies should improve their reporting around inclusivity, implementation, and evaluation.

The main strength of this review is the provision of a contemporary, global, synthesised list of dementia research priorities, which can be used to influence policy and research funding. The review has some limitations. Firstly, each included study produced a distinct list of priorities which varied in length, giving studies with a high number of priorities a higher weighting. In future, a set limit of priorities such as a ‘top 10’ could be applied to all papers. Secondly, this review was restricted to two languages, and relevant studies published in other languages may have been missed as a result. Some priority setting exercises may have been promoted using methods other than publication in a peer review journal. However, our search of specialist society websites did not find unpublished priorities.

Research prioritisation provides the opportunity to direct funding and resources to areas of key stakeholder priority. Despite differences by healthcare setting, there is a clear perceived need for more research around care‐givers and support. Priorities are shaped by the stake‐holder groups included and future exercises should consider including people from differing geographical regions and at differing stages of the dementia journey.

## CONFLICTS OF INTEREST

The authors have no conflicts to declare.

## Supporting information

Supporting Information S1

Figure S1

Figure S2

## Data Availability

Data from the review are available upon reasonable request to the corresponding author.
