# Supplementary material for: Comparing international dementia research priorities—Systematic review
Source: Int J Geriatr Psychiatry. 2022 Nov 3;37(12):10.1002/gps.5836. doi: 10.1002/gps.5836 (PMC9828247; doi:10.1002/gps.5836)
Supplement: Supplementary file 1 — Supporting Information S1 [file GPS-37-0-s001.docx]

**Supplementary information**

**Supplementary information 1: Search strategy.**

The primary concepts of dementia and research prioritisation were established; validated search syntax related to both was used and refined. Refined search terms included {dement*/and research*.tw.} {cognit*/ and research*.tw.} {alzheimer*/and research*.tw.} {priorit* AND research AND dementia} {priorit* AND alzheimer* AND research.tw.} {cognit* AND priorit* AND research} {uncertaint* AND dement*.tw.} {uncertaint* AND alzheimer*.tw.} {priorit*/ and uncertain.tw.*}.

**Supplementary information 2: tables.**

|  |  | Shah 2016 | Leroi 2019 | Armstrong 2020 | Bethell 2018 | Kelly 2015 | Law 2011 | Emrich-Mills 2019 | Hirt 2020 | Porock 2015 | Stolee 2011 |
| --- | --- | --- | --- | --- | --- | --- | --- | --- | --- | --- | --- |
|  | **Context and scope** |  |  |  |  |  |  |  |  |  |  |
| 1 | Define geographical scope |  |  |  |  |  |  |  |  |  |  |
| 2 | Define health area, field, focus |  |  |  |  |  |  |  |  |  |  |
| 3 | Define end-users of research |  |  |  |  |  |  |  |  |  |  |
| 4 | Define the target audience of the priorities |  |  |  |  |  |  |  |  |  |  |
| 5 | Identify the broad research area |  |  |  |  |  |  |  |  |  |  |
| 6 | Identify the type of research question |  |  |  |  |  |  |  |  |  |  |
| 7 | Define the time frame |  |  |  |  |  |  |  |  |  |  |
|  |  |  |  |  |  |  |  |  |  |  |  |
|  | **Governance and team** |  |  |  |  |  |  |  |  |  |  |
| 8 | Describe selection of the leadership and management team |  |  |  |  |  |  |  |  |  |  |
| 9 | Describe the characteristics of the team, and the networks they represent |  |  |  |  |  |  |  |  |  |  |
| 10 | Describe any training or experience in priority setting |  |  |  |  |  |  |  |  |  |  |
|  |  |  |  |  |  |  |  |  |  |  |  |
|  | **Framework for priority setting** |  |  |  |  |  |  |  |  |  |  |
| 11 | State the framework used (if any) |  |  |  |  |  |  |  |  |  |  |
|  |  |  |  |  |  |  |  |  |  |  |  |
|  | **Stakeholders or participants** |  |  |  |  |  |  |  |  |  |  |
| 12 | Define the inclusion criteria for stakeholders involved in priority-setting |  |  |  |  |  |  |  |  |  |  |
| 13 | State the strategy or method for identifying and engaging stakeholders |  |  |  |  |  |  |  |  |  |  |
| 14 | Indicate the number of participants and/or organisations involved |  |  |  |  |  |  |  |  |  |  |
| 15 | Describe the characteristics of stakeholders |  |  |  |  |  |  |  |  |  |  |
| 16 | State if reimbursement for participation was provided |  |  |  |  |  |  |  |  |  |  |
|  |  |  |  |  |  |  |  |  |  |  |  |
|  | **Identification and collection of research priorities** |  |  |  |  |  |  |  |  |  |  |
| 17 | Describe methods for collecting priorities from stakeholders |  |  |  |  |  |  |  |  |  |  |
| 18 | Describe methods for collating and categorizing priorities |  |  |  |  |  |  |  |  |  |  |
| 19 | Describe methods and reasons for removing priorities |  |  |  |  |  |  |  |  |  |  |
| 20 | Describe methods for refining or translating priorities into research topics or questions |  |  |  |  |  |  |  |  |  |  |
| 21 | Describe methods for checking whether research questions or topics have been answered |  |  |  |  |  |  |  |  |  |  |
| 22 | Describe number of research questions or topics |  |  |  |  |  |  |  |  |  |  |
|  |  |  |  |  |  |  |  |  |  |  |  |
|  | **Prioritisation of research topics/questions** |  |  |  |  |  |  |  |  |  |  |
| 23 | Describe methods and criteria for prioritising research topics or questions |  |  |  |  |  |  |  |  |  |  |
| 24 | Provide reasons for excluding research topics/questions |  |  |  |  |  |  |  |  |  |  |
|  |  |  |  |  |  |  |  |  |  |  |  |
|  | **Output** |  |  |  |  |  |  |  |  |  |  |
| 25 | Specificity of research priorities are clear |  |  |  |  |  |  |  |  |  |  |
|  |  |  |  |  |  |  |  |  |  |  |  |
|  | **Evaluation and feedback** |  |  |  |  |  |  |  |  |  |  |
| 26 | Describe how the process of prioritization was evaluated |  |  |  |  |  |  |  |  |  |  |
| 27 | Describe the approach for feeding back priorities to stakeholders and/or to the public; and how feedback was addressed and integrated |  |  |  |  |  |  |  |  |  |  |
|  |  |  |  |  |  |  |  |  |  |  |  |
|  | **Implementation** |  |  |  |  |  |  |  |  |  |  |
| 28 | Outline the strategy or action plans for implementing priorities |  |  |  |  |  |  |  |  |  |  |
| 29 | Describe evaluation of impact |  |  |  |  |  |  |  |  |  |  |
|  |  |  |  |  |  |  |  |  |  |  |  |
|  | **Funding and conflict of interest** |  |  |  |  |  |  |  |  |  |  |
| 30 | State sources of funding |  |  |  |  |  |  |  |  |  |  |
| 31 | Outline the budget and/or cost |  |  |  |  |  |  |  |  |  |  |
| 32 | Provide declaration of conflict of interest |  |  |  |  |  |  |  |  |  |  |

Supplementary Table 1. Completed REPRISE checklist for studies included in the review^35^. Components marked green represent high quality practice whilst components marked in red represent low quality practice. Amber indicates moderate quality.

**Supplementary information 3: figure captions.**

Supplementary Figure 1. PRISMA flow diagram for studies included in this review.

Supplementary Figure 2. Top three priorities (where applicable) for each category. Priorities were identified from included studies due to increased prominence and frequency.
