# Supplementary figures and images for: Comparing international dementia research priorities—Systematic review
Source: Int J Geriatr Psychiatry. 2022 Nov 3;37(12):10.1002/gps.5836. doi: 10.1002/gps.5836 (PMC9828247; doi:10.1002/gps.5836)

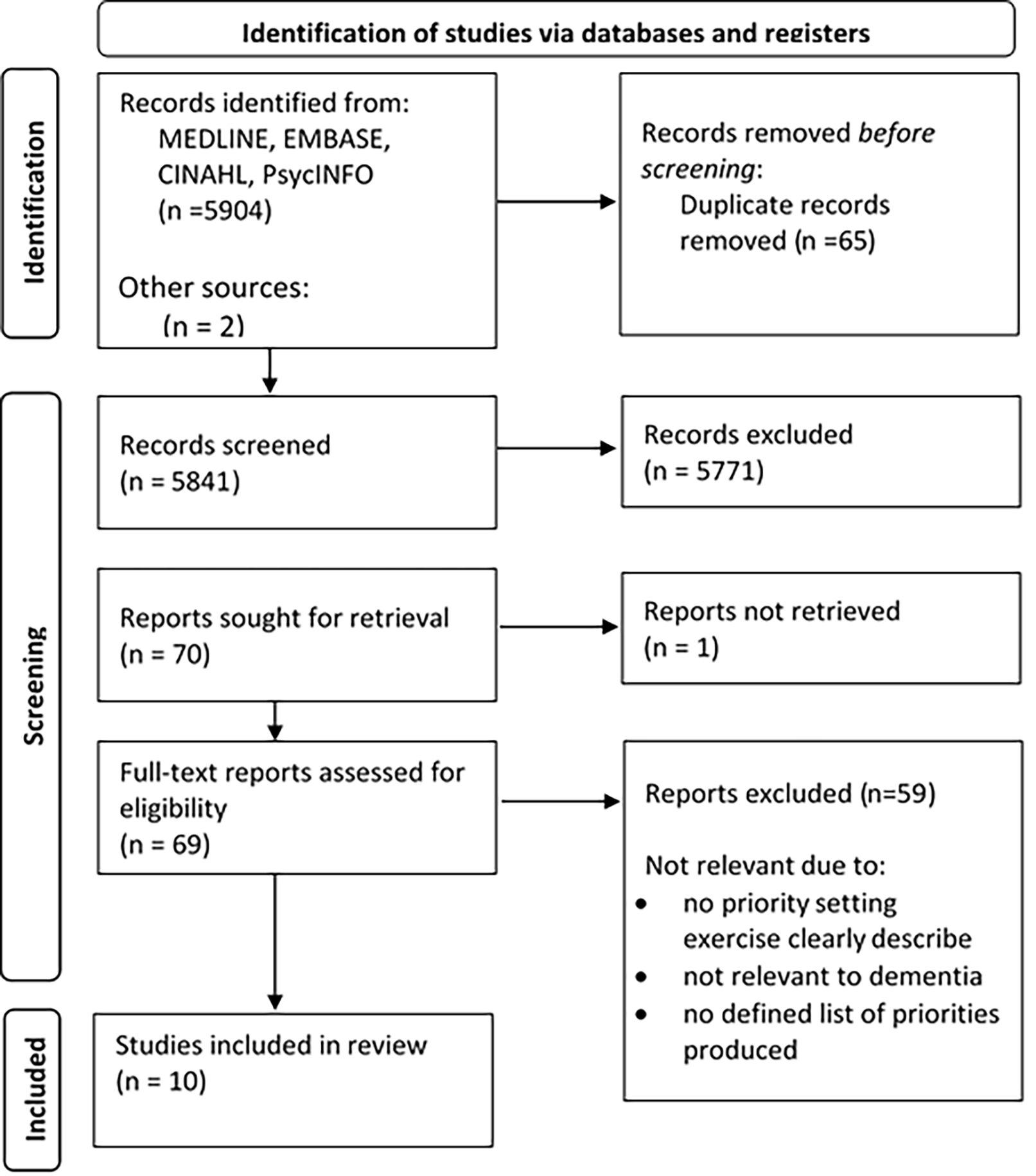

Supplement: Supplementary file 2 — Figure S1 [file GPS-37-0-s002.tif]

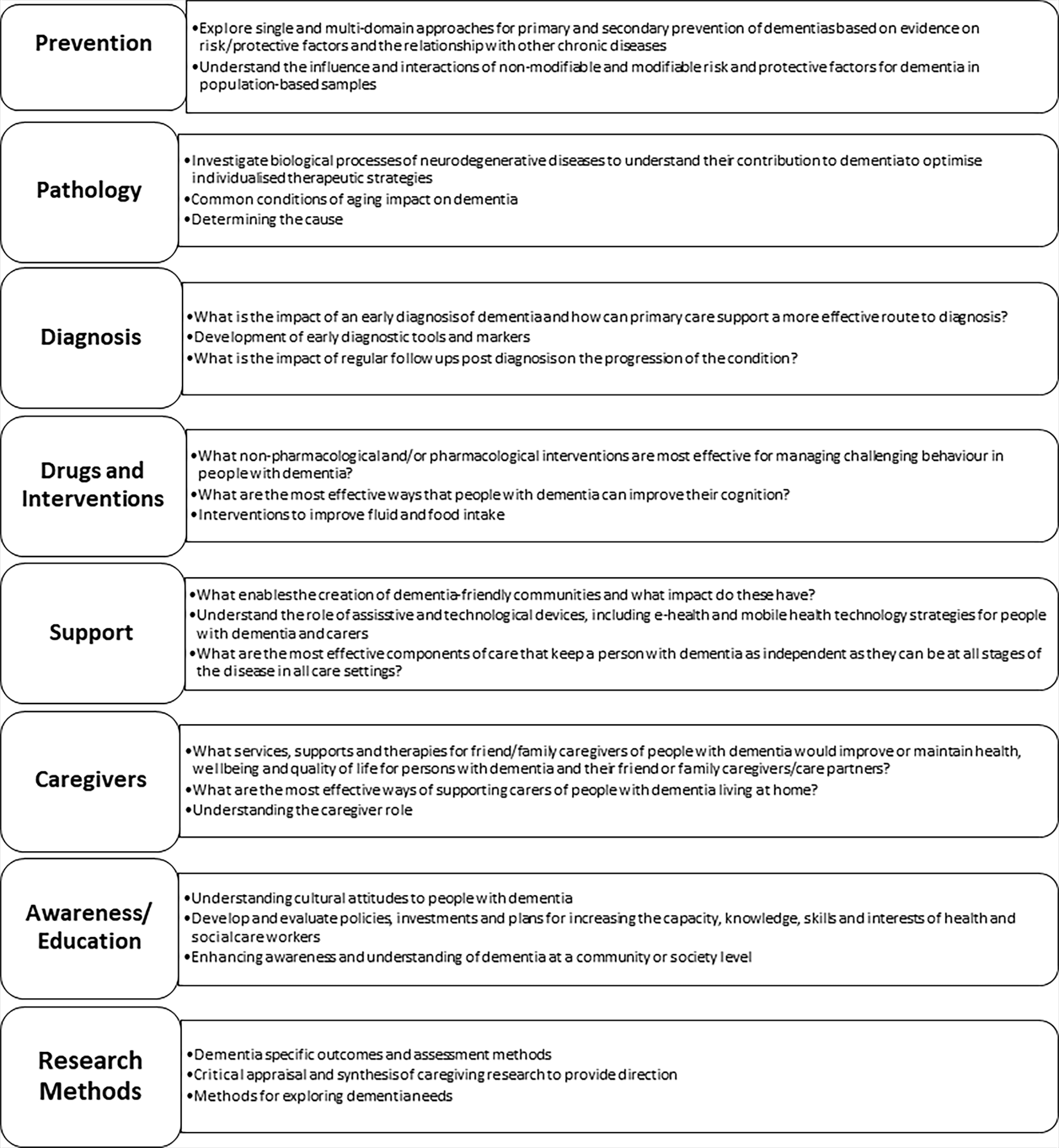

Supplement: Supplementary file 3 — Figure S2 [file GPS-37-0-s003.tif]
